# 2. MICROCIRCUITS, MACROCIRCUITS, AND CORTICOL DYSFUNCTION IN SCHIZOPHRENIA: A COMPUTATIONAL AND TRANSLATIONAL NEUROSCIENCE PERSPECTIVE

**DOI:** 10.1093/schbul/sby014.001

**Published:** 2018-04-01

**Authors:** John Krystal

**Affiliations:** Yale University School of Medicine

## Abstract

**Overall Abstract:** Computational neuroscience may be a critical component of the effort to understand how cortical micro- and macro-circuits support behavior and express the symptoms of neuropsychiatric disorders. This presentation will present an update on an ongoing interdisciplinary effort to understand the role of compromised glutamate synaptic signaling, particularly related to the NMDA glutamate receptor, for the pathophysiology of schizophrenia. This presentation will draw on studies in animal models, healthy humans, and schizophrenia patients. It will draw parallels between the effects of the NMDA receptor antagonist, ketamine, and working memory impairment and abnormalities in cortical functional connectivity in schizophrenia. In so doing, it will highlight examples where computational approaches have affirmed hypotheses arising from experimental work or contributed new predictions that could be tested experimentally. Lastly, it will illustrate a prediction about novel therapeutics for schizophrenia that are embedded in an emerging developmental model for this disorder.

